# Extrapolation of Survival Curves from Cancer Trials Using External Information

**DOI:** 10.1177/0272989X16670604

**Published:** 2016-09-29

**Authors:** Patricia Guyot, Anthony E. Ades, Matthew Beasley, Béranger Lueza, Jean-Pierre Pignon, Nicky J. Welton

**Affiliations:** School of Social and Community Medicine, University of Bristol, Bristol, UK (PG, AED, NJW); Mapi, Houten, the Netherlands (PG); Bristol Haematology and Oncology Centre, Bristol, UK (MB); Gustave Roussy, Université Paris-Saclay, Service de biostatistique et d’épidémiologie / Ligue Nationale Contre le Cancer meta-analysis plateform, F-94805 Villejuif, France (BL, JP); Université Paris-Saclay, Univ. Paris-Sud, UVSQ, CESP, INSERM, F-94085 Villejuif, France (BL, JP)

**Keywords:** cost-effectiveness analysis, survival analysis, restricted cubic splines, external data, extrapolation

## Abstract

**Background:** Estimates of life expectancy are a key input to cost-effectiveness analysis (CEA) models for cancer treatments. Due to the limited follow-up in Randomized Controlled Trials (RCTs), parametric models are frequently used to extrapolate survival outcomes beyond the RCT period. However, different parametric models that fit the RCT data equally well may generate highly divergent predictions of treatment-related gain in life expectancy. Here, we investigate the use of information external to the RCT data to inform model choice and estimation of life expectancy. **Methods:** We used Bayesian multi-parameter evidence synthesis to combine the RCT data with external information on general population survival, conditional survival from cancer registry databases, and expert opinion. We illustrate with a 5-year follow-up RCT of cetuximab plus radiotherapy v. radiotherapy alone for head and neck cancer. **Results:** Standard survival time distributions were insufficiently flexible to simultaneously fit both the RCT data and external data on general population survival. Using spline models, we were able to estimate a model that was consistent with the trial data and all external data. A model integrating all sources achieved an adequate fit and predicted a 4.7-month (95% CrL: 0.4; 9.1) gain in life expectancy due to cetuximab. **Conclusions:** Long-term extrapolation using parametric models based on RCT data alone is highly unreliable and these models are unlikely to be consistent with external data. External data can be integrated with RCT data using spline models to enable long-term extrapolation. Conditional survival data could be used for many cancers and general population survival may have a role in other conditions. The use of external data should be guided by knowledge of natural history and treatment mechanisms.

In randomized controlled trials (RCTs) reporting survival outcomes, progression-free survival and overall survival are generally graphically displayed with the Kaplan-Meier method (KM)^1^ and analyzed by Cox Regression.^2^ However, neither method is sufficient for the purposes of Cost Effectiveness Analysis (CEA), because they do not provide a model that can be extrapolated beyond the RCT period. Instead, parametric survival models are required to estimate expected survival.^3^ Models most often used in submissions to the National Institute for Health and Care Excellence (NICE) have a parametric model, such as Exponential, Weibull, Log-logistic, or Log-normal^4-5^ distributions, for the control arm, and a constant hazard ratio (proportional hazards) to predict the treatment arm.^3^ Parametric distributions differ in their flexibility: the exponential requires a constant hazard, the Weibull has monotonically increasing or decreasing hazards, while the log-normal and log-logistic allow for “bowl” or “hat” shaped hazards.^1^ Sometimes, the proportional hazard assumption is relaxed by fitting an accelerated failure time (AFT) model, or by fitting unrelated models to each arm, also known as Fitted Separately to Each Arm (FSEA).^3^ Model selection is not straightforward, and models that fit equally well to the observed RCT data may give very different estimated mean survival gains.^6^ This is due to mean survival being very sensitive to the tails of the survival distribution, which are usually not captured within RCT follow-up periods.

There is a well-recognized need to improve extrapolation of survival data for use in the context of cost-effectiveness analysis^7^ and there have been several attempts to do this using data external to the trial. One approach has been to use external data to inform the choice of parametric model to extrapolate the trial data.^8-11^ Other investigators have replaced the control arm by external data, with^12-14^ or without^15-17^ adjustment, while maintaining the treatment effect from the trial. Most methods using data external to the trial require a process of matching the trial and external populations,^18^ but investigators have differed in the relative weight given to each, and in how uncertainty in each source of information is reflected in the final model.

This paper presents a method for using external information to extrapolate survival curves in the specific area of cancer trials. Cancer is an interesting and important area in this respect for several reasons. First, there is a wealth of information about long-term survival from cancer registries. Second, the difficulties in choosing parametric models for extrapolation is well-understood,^4^ and, in the UK, these have contributed to a series of controversies in decisions as to which new cancer treatments should be used in the National Health Service.^19-21^

A trial comparing radiotherapy plus cetuximab (Erbitux)^22^ to radiotherapy alone for head and neck cancer patients is used to illustrate our approach. We consider two types of external information to inform extrapolation of the control arm: population data on overall survival of an age- and sex-matched cohort, and cancer registry information on conditional survival of a matched cohort of head and neck cancer patients. A third external source is information on the relative treatment effect, derived from an analysis of the literature and treatment mechanisms.

We begin with a description of the illustrative dataset and, to motivate the paper, we compare the performance of a series of standard parametric survival models on goodness of fit and predicted gain in life expectancy. We then briefly review what is known about the clinical epidemiology of head and neck cancer and the mechanisms underlying treatment with radiotherapy and cetuximab, and provide some preliminary analyses of data from cancer registries and recent meta-analyses, which justify our use of conditional survival data and the external information on treatment effect. The statistical methods are presented in outline, with further details and WinBUGS code appearing in a Web Appendix. In the discussion section, we consider the general properties of our approach compared to previous uses of external data in the literature, and consider the generalizability of the methods to other cancers, and to extrapolation of survival curves more widely.

## Motivating Example

Bonner and others^22^ conducted an RCT to compare radiotherapy plus cetuximab v. radiotherapy alone, with overall survival recorded over a 5-year follow-up period. We have used a method^23^ that accurately reconstructs the life-table data from the Kaplan-Meier curves published in that paper. These data are used throughout the paper and are shown in the Web Appendix.

In the analyses that follow, we consider how to extrapolate both arms in order to inform a potential CEA of cetuximab, aimed at a target population represented by the Bonner trial. The trial was carried out between 1999 and 2002 on treatment-naïve patients in several countries. The patient sample was 80% male with median age of 57 years, range 34 to 83 years. The most common site of primary tumors was oropharynx (59.5%), followed by larynx (25.5%) and hypopharynx (15%). All patients were classified as stage III or IV. Other factors of potential clinical and cost-effectiveness relevance are the epidermal growth factor receptor (EGFR) status,^22^ the performance status, Karnofsky or ECOG (Eastern Cooperative Oncology Group), and the severity of comorbidities.^24-25^

### Results with Standard Survival Models without External Data

**Figure 1:**
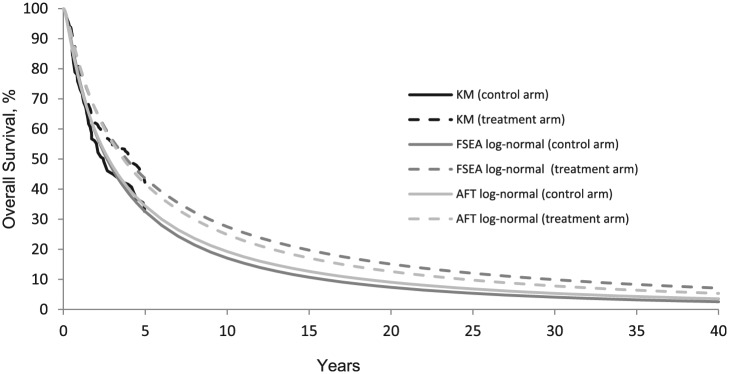
FSEA and AFT Log-normal models compared with Kaplan-Meier curves. KM, Kaplan-Meier; FSEA: Fitted Separately to Each Arm, PH: proportional hazards; AFT, accelerated failure time.

Table 1 shows Deviance Information Criteria (DIC),^26^ posterior mean deviance, $\bar{D}$, and gain in life expectancy predicted by 12 commonly used parametric survival models. $\bar{D}$ measures the overall model fit, whereas the DIC is a composite measure of fit and complexity useful to identify the most parsimonious model, trading off fit and complexity. Models with lower $\bar{D}$ and DIC are preferred although differences less than 3 (or even 5)^27^ are not considered important. Models with similar goodness of fit generate very different estimates of life expectancy gain (Table 1). Visually, the AFT and FSEA Log-normal models have an almost identical fit to the KM curves (Figure 1) and similar DIC (Table 1), but the predicted gain in life expectancy due to cetuximab is 80.4 months for the FSEA and only 32.3 months for AFT. Similarly, the Generalized Gamma^28^ AFT gives similar DIC to the Log-normal FSEA; however, the predicted survival gain is only 13.9 months, nearly 6 times lower. The log-normal and Generalized Gamma models present the best fit to the data according to the $\bar{D}$ and DIC values.

**Table 1:** Model Fit Statistics (Posterior Mean Deviance, $\bar{D}$, and Deviance Information Criteria, DIC) and Estimated Differences in Life Expectancy between the Two Arms of Bonner^22^ RCT for Different Survival Models, With and Without External Data

	Total $\bar{D}$	Total DIC	Gain in Life Expectancy due to Cetuximab (Months)	
			Point Estimate	95% CrI
***Standard parametric models, no external data***				
2-parameter Gamma FSEA	2,343	2,345	21.0	(2.6; 44.5)
2-parameter Gamma AFT	2,342	2,345	18.0	(1.7; 36.9)
Weibull FSEA	2,341	2,344	23.3	(0.7; 54.5)
Weibull PH	2,341	2,343	19.4	(1.6; 40.9)
Exponential FSEA	2,342	2,342	17.0	(2.1; 33.4)
Exponential PH	2,342	2,343	17.0	(2.0; 33.4)
Log-logistic FSEA	2,322	2,325	195.5	(-6895.0; 6860.0)
Log-logistic AFT	2,322	2,325	82.5	(-5.7; 487.8)
Log-normal FSEA	2,311	2,314	80.4	(2.0; 237.0)
Log-normal AFT	2,313	2,316	32.3	(-3.1; 78.6)
Generalized Gamma FSEA	2,308	2,313	50.9	(-19.2; 179.4)
Generalized Gamma AFT	2,310	2,313	13.9	(-4.3; 48.2)
***Standard parametric models with external data***				
Log-normal AFT, with general population data	2,327	2,329	32.4	(14.11; 55.73)
Generalized Gamma FSEA with general population data	2,317	2,322	31.7	(-26.4; 162.3)
***Spline model with external data***				
Splines with general population survival, conditional and relative treatment effect	2,303	2,306	4.7	(0.4; 9.1)

CrI: Credible Interval; DIC: Deviance Information Criterion; FSEA: Fitted Separately to Each Arm; PH: Proportional Hazards; AFT: Accelerated Failure Time.

For each parameter, or log parameter if the parameter was required by definition to be positive, the prior was assumed to follow a normal distribution with a mean of 0 and a variance of 1000.

## Clinical Epidemiology of Head and Neck Cancer and Mechanism of Treatments

Head and neck cancer usually displays a rapidly increasing mortality over the first 36 months from diagnosis, which then levels off.^29^ Survival rates differ markedly according to the site of the cancer. Cancer of the larynx is typically diagnosed earlier than cancers of the hypopharynx and oropharynx, and patients usually have a better chance of surviving their cancer.^24-25, 29^ Longer-term, registry-based studies from the US and Netherlands^30,31^ show that relative survival of head and neck cancer stabilizes after five or six years. Hence, UK guidance advises regular examination of the neck during the first two years after treatment, and discharge from routine follow-up after five years.^32^ Subsequently, patients can be considered as essentially “cured” from their head and neck cancer; although they continue to experience excess mortality due to risk factors associated with head and neck cancer, such as alcohol abuse, tobacco and Human Papillomavirus (HPV).^33, 34^ According to the above registry studies, this excess mortality persists for at least 15 years.

**Figure 2:**
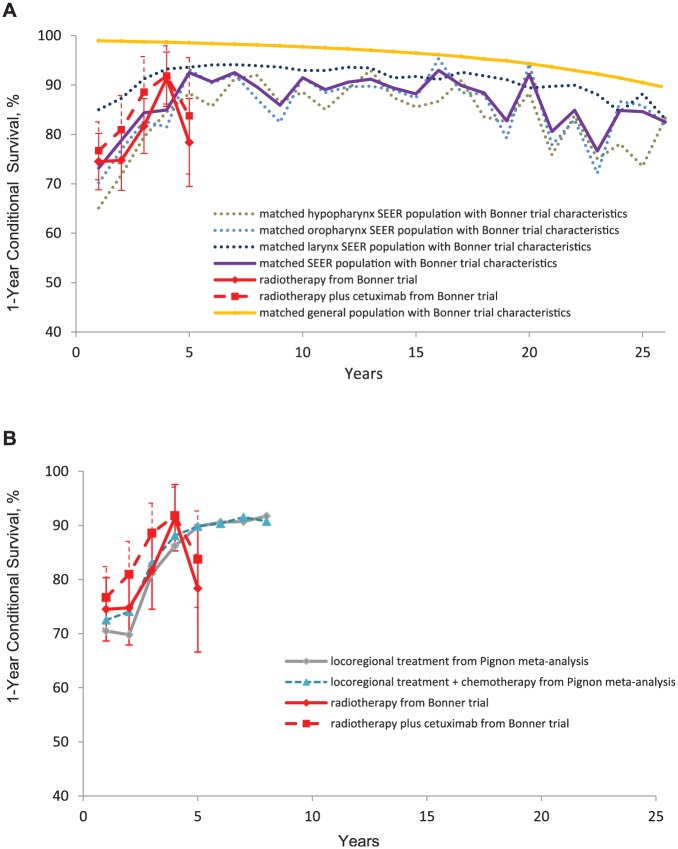
1-year conditional survival predicted in Bonner 2006 and in the Surveillance, Epidemiology, and End Results (SEER) database (A). 1-year conditional survival predicted in Bonner 2006 and in Pignon 2009 (B).

To confirm the relevance of these results in the present context, we constructed a cohort of cancer patients matched to the Bonner trial population for age, gender, cancer site, and date of diagnosis^35^ using registry data from the Surveillance Epidemiology and End Results (SEER) database.^36^ One-year conditional survival in this cohort is shown alongside conditional survival in the trial (Figure 2A). The results accord closely with previous work reported above, and show the close agreement of trial and SEER data. Also shown is the conditional survival of an age- and sex- matched general population cohort based on US survival statistics.^37^ (See Web Appendix A and B details of how these matched cohorts were constructed). This confirms that conditional relative survival remains approximately constant and less than one for at least 20 years after the initial 5 years from diagnosis.

Cetuximab is administered over a single 8-week period concurrently with radiotherapy. Its mode of action is to enhance the effect of radiotherapy,^38^ by increasing the proportion of cancer cells that are sensitive to radiotherapy. For this reason, the time course of the effect of cetuximab can be expected to be the same as the time course of the effect of radiotherapy. Similarly, we would expect that the effect of both therapies in reducing head and neck cancer mortality should be limited to the initial 5 or 6 years during which mortality due to the head and neck cancer predominantly occurs.

To further check our interpretation of the clinical literature on head and neck cancer, we undertook additional analyses of data incorporated in the Pignon meta-analysis comparing loco-regional treatment (radiotherapy and surgery plus postoperative radiotherapy) v. the same loco-regional treatment plus chemotherapy.^39-40^ Five-year overall survival rates in the meta-analysis control arm and the Bonner control arm were very close (*P* = 0.74), in spite of some differences in stage distribution and site. Further, this independent source of data confirms the same pattern of conditional mortality as that of previous literature (Figure 2B): a rising conditional mortality over the first 5 years, reflecting an increasing proportion of deaths from causes other than head and neck cancer, followed by stable conditional mortality in years 6 to 8.

Also, confirming our understanding of the treatment mechanisms, the advantage of chemotherapy in conditional survival can be seen over years 1 to 5, but disappears after that. To our knowledge, there is currently no evidence associating cetuximab with benefits, or toxicities, after five years.

Based on these findings, we make the following three assumptions

1. *General population survival: S*urvival in the control arm of the trial will remain less than survival in the matched general population cohort over the entire time horizon of the CEA model.
2. *Conditional survival*: One-year conditional survival in the trial control arm will converge to that of a matched cancer cohort, five or six years after diagnosis.
3. *Relative Treatment effect*: The hazard ratio changes over time as a smooth function with one turning point (expected to decrease initially and then increase). The hazard ratio is assumed to begin at 1 year, when treatment begins, and return to unity by 6 years.

## Statistical Methods

We begin by illustrating the impact of incorporating external data constraints on parametric survival models. We present results for the AFT Log-normal and the FSEA Generalized Gamma models. These were chosen as the most compatible with the RCT data, as indicated by DIC and $\bar{D}$ (Table 1), and because they varied in flexibility (number of parameters). A similar issues arose with all of the parametric survival models.

As a more flexible alternative, cubic spline functions were chosen to model the relationship between log cumulative hazards and log-time.^41,55^ These are cubic polynomials fitted to successive sets of two points, or internal knots, in a way that guarantees they are continuously differentiable. Further details are given in Web Appendix C. Two spline models were created on the log cumulative hazard scale, one for the control arm and one for the additional relative effect of treatment.

Boundary knots were placed at the extreme ends of the data. We also specified one internal knot in the RCT segment of the data, and another internal knot in the period between the end of the trial (5 years) and the end of the external data (40 years). The knots were placed in the mid-point on the log-time scale.

External information was added incrementally: data on general population and the SEER database for the control arm were first included separately, then together, and finally information was also added on the hazard ratio.

External information was introduced by specifying the relationship between the parameters estimated by the external information, and the parameters of the survival distributions, whether these were parametric or spline functions. This was implemented by writing the likelihoods for the external data in terms of the parameters of the (extrapolated) survival model that also gives the likelihood for the trial data. In this way, a single survival model is estimated from all sources of data (RCT and external) simultaneously.

### Estimation

Estimation was carried out by Bayesian Markov Chain Monte Carlo (MCMC) simulation using WinBUGS^42^ and WBDev.^43^ Observed and fitted survival, conditional survival, and relative hazard ratios were visually compared. $\bar{D}$ and DIC statistics were computed within the MCMC simulation and transformed using a constant to the scale of the parametric models to allow comparisons.^10^ These statistics were recorded, separately for the RCT data and each type of external data. This allowed us to compare the model fit to the different data types. The threshold for choosing one model over another was a 5-point difference in the DIC^27^ on the RCT data. Details regarding choice of initial values,^56^ convergence checks, burn-in period, and posterior sampling are given in Web Appendix D.

### Introducing External Data on Absolute Survival in the General Population

The age- and gender-matched general population is expected to have overall survival, $S_{\mathrm{GP}}(t)$, that is no lower than survival $S_{0,\mathrm{RCT}}(t)$ in the RCT control arm at any time t. We cannot put constraints directly on $S_{0,\mathrm{RCT}}(t)$, because this is a complex function of the survival model parameters. Instead, we introduced the external data in a way that imposes the constraint on the extrapolated curves. We do this at a single time point at 40 years, when the matched cohort had $r_{\mathrm{GP}}(40)=1,660$ survivors out of a denominator of $n_{\mathrm{GP}}(40)=158,858$ persons in the matched cohort at time 0 (see Web Appendix A), corresponding to 40-year survival 1.045% [95CrL: 1.036; 1.054]. Assuming a binomial distribution for this data, we implemented the belief that survival in the control arm of the RCT at 40 years, $S_{0,\mathrm{RCT}}(40)$, is no better than that in the matched general population, $S_{0,\mathrm{RCT}}(40)\leq S_{\mathrm{GP}}(40)$, by giving a Binomial likelihood for the general population data:

$$\begin{array}{c} r_{\mathrm{GP}}(40)\sim \mathrm{Binomial}\;(S_{\mathrm{GP}}(40),n_{\mathrm{GP}}(40))\\ \mathrm{where}\;S_{\mathrm{GP}}(40)\;\mathrm{is}\;\mathrm{constrained}\;\mathrm{to}\;\mathrm{be}\\ \mathrm{greater}\;\mathrm{than}\;S_{0,\mathrm{RCT}}(40)\;\mathrm{so}\;\mathrm{that}\\ S_{\mathrm{GP}}(40)=S_{0,\mathrm{RCT}}(40)+\beta ,\;\beta >0 \end{array}$$

Additional constraints are required to ensure that $S_{\mathrm{GP}}(40)$ lies in the interval 0,1.

### Introducing External Data from SEER on Conditional Survival

We assumed that the RCT control arm population 1-year conditional survival at time *t* conditional on being alive at time (*t*-1), $CS_{0,\mathrm{RCT}}(t|t-1)$, is no different to the matched SEER population conditional survival, $CS_{\mathrm{SEER}}(t|t-1)$, from 6 years onwards until 26 years (last time point available in the SEER database). The methods for calculating the numbers of person alive and at risk in the SEER population between time *t* and *t*– 1, respectively $r_{\mathrm{SEER}}(t|t-1)$ and $n_{\mathrm{SEER}}(t|t-1)$, are shown in the Web Appendix B, along with the data itself. Assuming a binomial likelihood for 1-year conditional survival probabilities from the SEER population, we implemented this by specifying:

$$\begin{array}{c} r_{\mathrm{SEER}}(t|t-1)\sim \mathrm{Binomial}(CS_{\mathrm{SEER}}(t|t-1),n_{\mathrm{SEER}}(t|t-1))\\ \mathrm{where}\;CS_{\mathrm{SEER}}(t|t-1)\;\mathrm{is}\;\mathrm{constrained}\\ \;\mathrm{to}\;\mathrm{be}\;\mathrm{equal}\;\mathrm{to}\;CS_{0,\mathrm{RCT}}(t|t-1)\;\mathrm{so}\;\mathrm{that}\\ CS_{\mathrm{SEER}}(t|t-1)=CS_{0,\mathrm{RCT}}(t|t-1),\;6\leq t\leq 26\;\mathrm{years} \end{array}$$

### Introducing Both General Population Survival Data and Conditional Survival Data

When adding the SEER data on conditional survival (Equation [2]), we observed that the 1-year conditional estimated survival curve for the RCT population crossed that from the general population at 33 years. To avoid conflict between the two sources of external data, we assumed that 1-year conditional survival in the control arm of the RCT was no different to that in the general population at a time point. In addition to equation (2) we therefore specify:

$$\begin{array}{c} r_{\mathrm{GP}}(35|34)\sim \mathrm{Binomial}\;(CS_{\mathrm{GP}}(35|34),n_{\mathrm{GP}}(35|34))\\ \mathrm{where}\;CS_{\mathrm{GP}}(35|34)\;\mathrm{is}\;\mathrm{constrained}\\ \mathrm{to}\;\mathrm{be}\;\mathrm{equal}\;\mathrm{to}\;CS_{0,\mathrm{RCT}}(35|34)\;\mathrm{so}\;\mathrm{that}\\ CS_{\mathrm{GP}}(35|34)=CS_{0,\mathrm{RCT}}(35|34) \end{array}$$

### Introducing External Data on the Relative Treatment Effect

We expected that the hazard ratio is a smooth function with a monotonic decrease followed by a monotonic increase over years 0-5. This is captured by having a single knot in this interval. To reflect that there is some uncertainty about whether the hazard ratio returns exactly to 1, we introduced external data on the hazard ratio taking the value of 1 at time points t=6…35years, each with a standard error of 0.1. This was achieved using a normal likelihood:

$$HR(t)\sim N(\frac{h_{1,\mathrm{RCT}}(t)}{h_{0,\mathrm{RCT}}(t)},0.1^{2}),\;t=6\ldots 35\;\mathrm{years}$$

where $h_{0,\mathrm{RCT}}(t)$ and $h_{1,\mathrm{RCT}}(t)$are the hazards on the control and treatment arms of the RCT population, respectively.

Winbugs code and data are presented in Appendix D.

## Results

### Parametric Models with External Data on General Population Survival

**Figure 3:**
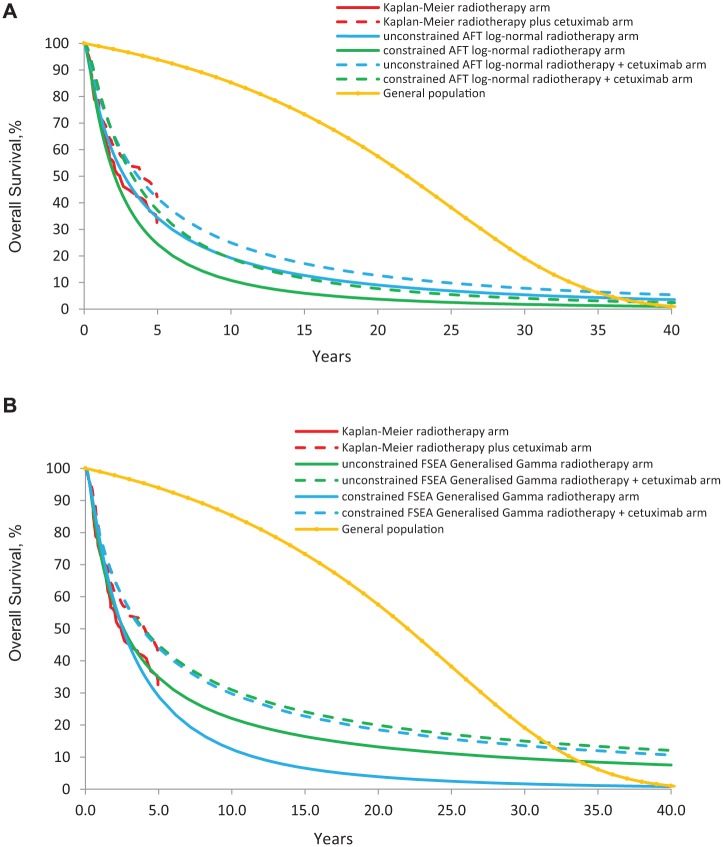
Overall survival predicted by KM, unconstrained AFT Log-normal, and AFT Log-normal constrained by general population data, using Equation 1 (A). Overall survival predicted by KM, unconstrained FSEA Generalized Gamma, and FSEA Generalized Gamma constrained by general population data, using Equation 1 (B). KM, Kaplan-Meier; FSEA, Fitted Separately to Each Arm; AFT, accelerated failure time.

Figure 3A shows estimated curves from an AFT 2-parameter Log-normal model for (i) the unconstrained model with no external information, (ii) the model constrained by overall survival in the general population (Equation [1]), together with the KM-curves. It is evident that the external data are far from compatible with the trial data under this model. The fit of the trial data deteriorates markedly with the inclusion of external data, with a DIC increasing from 2,316 to 2,329 and a $\bar{D}$ from 2,313 to 2,327 (Table 1). Similar results were obtained with the FSEA Generalized Gamma (Figure 3B). Neither of these distributions is sufficiently flexible to comply with both the RCT and the general population data (Equation [1]). The same problem arose with all of the parametric models reported in Table 1. Further external data cannot improve the flexibility of the parametric models, and so we do not present results including further external data.

### Spline Models

With restricted cubic splines with two internal knots, DIC results for the unconstrained model were between 2,304 and 2,306, depending on the sets of initial values. When the data on general population survival were added, the DIC was 2,304 (Table 2). Convergence of the MCMC simulations was not satisfactory for all parameters in the model, due to the lack of data between the end of the trial and year 40, making it impossible to identify spline parameters (see Web Appendix E). These numerical problems were overcome when further external constraints were incorporated.

**Table 2:** Global Goodness of Fit Statistics (Posterior Mean Deviance, $\bar{D}$, and Deviance Information Criteria, DIC) for the Internal and External Data Elements

	RCT Data		SEER Data		General Population Data		Relative Treatment Effect	
	$\bar{D}$	DIC	$\bar{D}$	DIC	$\bar{D}$	DIC	$\bar{D}$	DIC
Log logistic AFT using Equation [1]	2,327	2,329	NA	NA	10	11	NA	NA
Gen Gamma FSEA using Equation [1]	2,317	2,322	NA	NA	10	11	NA	NA
Splines, no external data used	2,300	2,307	NA	NA	NA	NA	NA	NA
Splines using Equation [1]	2,300	2,307	NA	NA	10	11	NA	NA
Splines using Equation [2]	2,302	2,307	96	98	NA	NA	NA	NA
Splines using Equation [2] and Equation [3]	2,302	2,307	96	97	11	12	NA	NA
Splines using Equation [2], Equation [3] and Equation [4]	2,303	2,306	97	98	11	12	−62	−60

When the external data on conditional survival was incorporated, the DIC value for the RCT data from the spline model was found equal to 2,307, very close to DIC without external data (Table 2). After the RCT period, the extrapolated trial curves were visually close to the SEER data (DIC value of 98). The SEER data fully identified the extrapolated control arm survival curve. The spline model therefore produced a survival estimate that was consistent with both RCT and all external evidence sources.

When conditional survival in the general population (Equation [3]) and SEER data (Equation [2]) were both applied, DIC for the RCT and the SEER data barely changed (Table 2). The survival curves estimated from the RCT and the general population now cross at 35 years after the start of the RCT. We could have extended this general population constraint by adding time points between 36 and 60 years. However, considering the low percentage of patients still alive 35 years after the start of the RCT (median age at randomization was 57 years), we did not try to incorporate more general population data.

**Figure 4:**
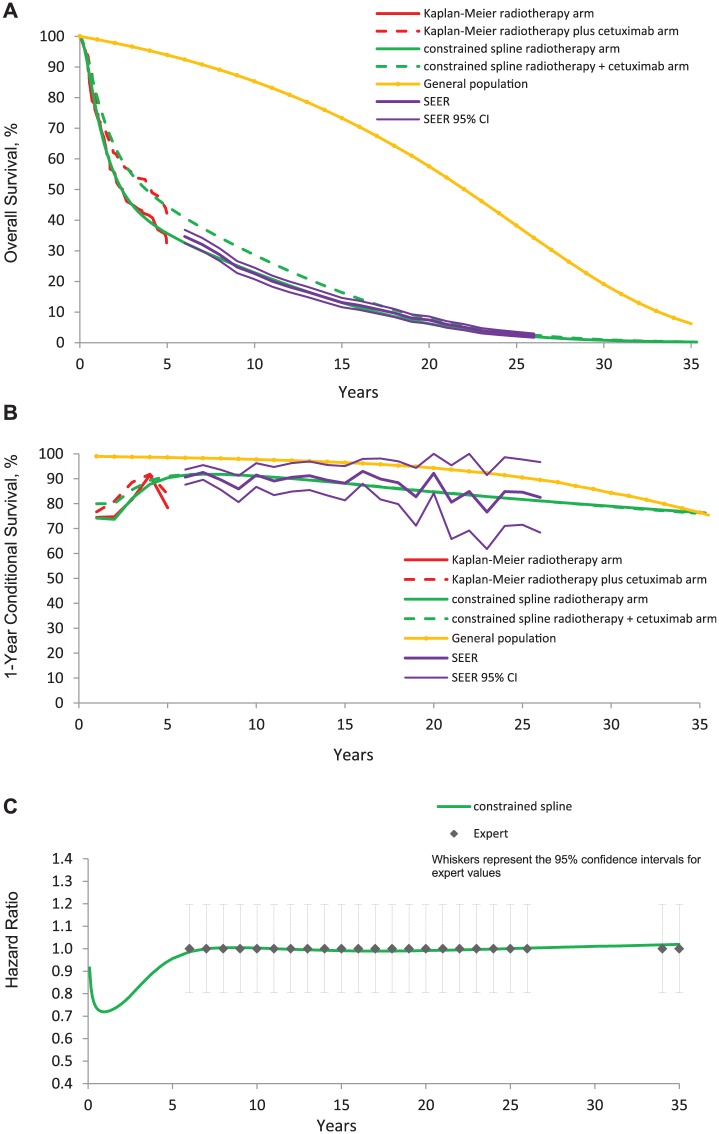
Overall survival predicted by KM, unconstrained splines, and splines constrained by general population data using Equation 3, SEER conditional survival data using Equation 2, and expert data using Equation 4 (A). 1-year conditional survival (B). Hazard ratio (C).

When the data on the hazard ratio was also added (Equation [4]), DIC for trial, SEER, and general population data again barely changed (Table 2). Figure 4 suggests a visually good fit of the spline models to each source of data. The estimates complied with the RCT data during the RCT period and with SEER, general population and expert data after the end of the RCT. Using the flexible spline models, we have therefore managed to incorporate all external data, without deterioration in model fit for any of the different evidence sources.

The life expectancy gain when all external data sources were incorporated was 4.7 months [95%CrL: 0.4; 9.1] (Table 1).

## Discussion

We begin this concluding section by reviewing the choice of survival function (splines, parametric models, and other options). We then compare our use of external data to previous work in the literature from a technical point of view, with a particular focus on uncertainty propagation. Third, we consider the modeling choices facing investigators using external data, and suggest extensions and sensitivity analyses that could be used in practical applications. Finally, we consider the particular type of external information used here, and what this implies about the generalizability of the methods to other cancers and other conditions.

### Survival Models

The study adds to a growing literature on the difficulties associated with extrapolation of survival curves using standard parametric models.^3, 6^ Even the models that fitted the RCT data best without external data fitted the trial quite poorly compared to spline models with external data. Indeed, although it seems a weak requirement that the extrapolated survival in a cancer trial control arm must be less than would be observed in the general population, for this dataset, it was easily strong enough to rule out the standard parametric curves. These distributions do not provide the possibility of “local” parameters: the behavior of the tails is strongly determined by data fit at the very beginning, and vice versa. It remains to be seen whether other flexible distributions, such as fractional polynomials,^43^ mixture distributions,^44-46^ or join-point techniques could represent valid alternatives to the splines, which we found gave rise to numerical problems in some contexts (see Web Appendix).

### Uncertainty Propagation

There are many examples of the use of external data on the general population^9,11,13^ and also the use of registries, meta-analyses or prospective observational studies of cancer cohorts.^8,10,12-13,15-17,47^ Many applications are tailored to specific circumstances and it is difficult to give an overall review. There are, however, marked differences in the way uncertainty is propagated. Our approach to the combination of trial and external data is an example of Bayesian multiple parameter evidence synthesis (MPES).^48^ Typically, vague priors are assigned to the basic parameters^49^ and information on parameters or functions of parameters is introduced by the data likelihoods. Simultaneous estimation from all these data sources ensures a coherent model that is consistent with all the evidence included, and that fully reflects the statistical uncertainty in the evidence for propagation into a cost-effectiveness model. We would also claim that, by using the highly flexible cubic splines, we have also appropriately accounted for uncertainty in the choice of survival model, conditional, of course, on our assumptions about the number of inflexions implied by the number of internal knots.

In terms of uncertainty propagation, we therefore believe our general approach has some clear technical advantages over other ways of using external data. For example, the use of external data to choose a parametric model^8-9^ fails to account for uncertainty in the model choice. Methods that “import” an estimate of the relative effect from the trial and overlay this on a model of the control arm, will misrepresent the uncertainty in the treatment effect estimate, because it was generated from a different model of the control arm. In our approach, we advocate all parameters are estimated simultaneously, using combined data from the RCT, the general population survival data, conditional survival data, and information on the relative treatment effect.

The main difficulty with importing a treatment effect estimate from a different model is that, in virtually every case, investigators have relied on a proportional hazard assumption. Based on a logical consideration of how treatments work, our view is that the proportional hazards assumption is highly implausible in many cancer treatment trials. Instead, the effect of a treatment on mortality risk can be expected to accelerate over an initial period, and then decelerate as non-cancer causes of death begin to predominate. Although we have made quite strong assumptions about the relative treatment effect, they are reasonably well-grounded in evidence and in theory, and far weaker than the routinely accepted proportional hazards assumption.

In view of the wild variation in estimated life expectancy gain between parametric models (Table 1), it is interesting to observe that the relative uncertainty in our final estimate of mean survival gain from the spline model with all sources of external data (4.7 months with 95%CrI 0.4 to 9.1 month) is commensurate with the log hazard ratio in the original Bonner trial:^22^ -0.30 (-0.56 to -0.03). Both estimates exclude the null effect by a narrow margin, and have a similar coefficient of variation (standard error divided by mean), based on the 95% intervals: 0.47 from the present study compared to 0.45 from the trial.

An earlier attempt to extrapolate survival in the Bonner trial appeared in the manufacturer’s submission to NICE.^47^ A cure model with a logistic link was used to model the survival of head and neck cancer patients. For the non-cured patients, a Log-Normal distribution was chosen. The non-cured fraction was extrapolated for both control and treated group, while subsequent mortality in the cured fraction was based on UK population data adjusted down using a hazard ratio derived from the Pignon meta-analysis.^40^ This generated an expected gain of 10.6 months. A credible interval was not published. This approach has several similarities to what we are advocating here, in assuming a time-limited treatment effect and stable conditional survival subsequently, but it relies on a specific parametric form for the cured fraction and fails to incorporate uncertainty in the model choice.

### Modeling Choices and Sensitivity Analyses

In constructing what is primarily a methodological exercise to demonstrate the feasibility of a method in principle, we have the luxury of not having to face the modelling choices that investigators in a real decision making context must confront. In this section, we consider how the present methods could be varied or extended to different target populations, and what kinds of sensitivity analyses might be required in practice.

While we believe our approach appropriately reflects statistical uncertainty in the combined data, as well as uncertainty in model choice, it cannot in itself propagate uncertainty about the relevance or applicability of the external evidence to the target population. Bayesian theory distinguishes strictly between the “subjective” prior and the “objective” data likelihood, but this overlooks the subjectivity in the interpretation of the data—specifically, the assumption that the data are providing unbiased estimates of the target parameters. For example, we assume that conditional survival is exactly the same in the target (trial) population as in the matched SEER cohort. Further analyses of SEER and other registries, such as those shown in Figure 2A, can be used to show how sensitive conditional survival might be to imbalances in cancer site mix, age, and date of diagnosis and Karnofsy score.

We used the US SEER data, and, hence, US population data, to supply external data on survival and conditional survival because of the comprehensiveness of this database. Decision makers in other jurisdictions would preferentially use locally relevant population and cancer registration data. If this was unavailable, or partially available, it might be still possible to use SEER data, perhaps making suitable adjustments. Note, however, that conditional survival or relative conditional survival is more likely to generalize across jurisdictions than overall survival. Alternatively, the similarity of SEER and other national cancer registries could be investigated to inform sensitivity analyses. Similarly, we assumed that the target population was identical to the trial population, which is frequently done in Health Technology Assessments, rather than replacing the control arm with appropriately matched external data that precisely represents the chosen target population. In this case, unless the external data is drawn from a cancer registry, it may still be necessary to use conditional survival register data in the way we have to extrapolate lifetime survival. Note that the same flexible spline approach we have proposed for the time course of the relative treatment effect can still be applied, whether the control arm is constructed from extrapolated trial data or based entirely on external data.

In a practical application, investigators would carry out a series of sensitivity analyses. Placement and number of internal knots in the spline function might seem obvious candidates, but general experience with splines is that the location of knots has little impact on estimates, whereas too few or too many knots may degrade the goodness-of-fit.^41, 50^ The strategy we have followed has been to place internal knots in the middle of the (log) range, to use the same points in all models, and to have just a single knot for each segment of data. These modeling choices avoid post hoc trawling, and are *a priori* the most easily defended. The choice of a single internal knot in the trial period is informed by our assumption that the hazard ratio would fall monotonically then rise, and that changes in conditional relative survival over time would be smooth. In a practical application, however, some sensitivity analysis around the placement of knots would be expected. One would also expect a sensitivity analysis around the assumption that the time at which the hazard ratio is assumed to return to unity.

The present analysis relied on a period-analysis^35^ of survival and conditional survival. This only partly allows for improvements over time in cancer survival. Another form of sensitivity analysis, or perhaps an extension to our approach, might consider a more sophisticated model of the registry data that allows for the continual improvement in cancer survival rates into the future. A further issue is the time between diagnosis and randomization. Patients in the Bonner^22^ trial would have been recruited soon after diagnosis, but the methods could be modified to allow for other scenarios.

More broadly, our construction of “external information” on the relative treatment effect is based on a whole series of assumptions about head and neck cancer and treatment mechanisms. This should not be regarded as a weakness of the analysis: similar assumptions would have to be made in any decision analysis concerned with cancer treatments. Investigators need to be explicit about their choice of external data and their interpretation of it, backing this up if possible with supporting analyses, such as those illustrated in Figure 2. The assumptions must be open to examination, debate, and sensitivity analyses, as in any other analysis of clinical or cost effectiveness.

### Limitations and Generalizability to Other Cancers and Conditions

Any use of data to make predictions requires subjective judgment about the relevance and applicability of the data. But among the limitations of the method, is the degree of reliance on subjective judgment about the clinical epidemiology of the condition and the treatment effect. “Uncertainty” regarding what are, in effect, structural assumptions, is difficult to express. Another limitation is the technical difficulty in fitting spline models, particularly in evidence-sparse situations. We have experienced this problem mainly when fitting models incrementally: the problem is much less acute when all sources of external data are included. A great deal could be learned from applying these methods to extrapolate a range of survival curves in other cancers.

Besides the uncertainty propagation properties of the method, which could be applied very generally, a more substantive contribution of the paper is the suggestion that relative conditional survival, based on cancer registers or meta-analyses of cancer trials, provides a relatively accurate way of extrapolating cancer trials. Studies of registry data from many countries shows that, while conditional survival is sensitive to age at diagnosis and stage at diagnosis, for many cancers, it tends to stabilize 5 to 8 years after diagnosis.^51-54^

We do not expect all cancers and all treatments to behave in precisely the same way as head and neck cancer. An examination of the available literature and registry data guided by clinical experts, alongside preliminary analyses, such as that in Figure 2, is a procedure that could be adopted in other circumstances. For example, cetuximab is usually administered in a single, relatively brief course: treatments involving a longer course, several courses, and other modes of treatment, like surgery or radiotherapy, might require a somewhat different approach. Similarly, if there was a suspicion of later toxicity, a sensitivity analysis could be readily constructed to allow for this.

In the present case, external data on general population survival did not represent much of a constraint on extrapolation, although it served to rule out a large number of commonly used models quite decisively. In the UK, where manufacturers submit evidence to NICE to obtain approval for the use of drugs in the NHS, uncertainty about which parametric model to apply when extrapolating cancer survival curves has, on several occasions, contributed to controversy that led eventually to an appeal process.^18-20^ The use of external information, in the way suggested above, both for extrapolating the control arm and the treatment effect, would contribute to obtaining better evidence-based estimates of gain in life expectancy.

## Supplementary Material

Supplementary material

Supplementary material

Supplementary material

Supplementary material

Supplementary material
