# Supplementary material for: Extrapolation of Survival Curves from Cancer Trials Using External Information
Source: Med Decis Making. 2016 Sep 29;37(4):353–66. doi: 10.1177/0272989X16670604 (PMC6190619; doi:10.1177/0272989X16670604)
Supplement: Supplementary material [file Extrapolation_Appendix_A_v10_online_supp.docx]

## Appendix A: Construction of a general population matched to the Bonner RCT population

General population survival data are available in the human mortality database (37). This database provides the number of persons exposed to the risk of death, the number of deaths and the probabilities of deaths, by age, from 0 to 109 years, gender, for years 1933-2009 and many countries.

The objective is to generate a general population cohort matched to the trial cohort for age at the onset of the trial and gender that differs from the trial population *only* in that it does not have head and neck cancer. $P_{a_{k}}$ , $P_{g}$ and $P_{c}$ are the proportions of RCT patients in each age group *a_k_*, gender *g* and country c. These figures are available in the Bonner publication. We assume that these factors are independent.

For age we used the published mean age and standard deviation, and divided the age into quartiles. The proportion of patients entering the trial at each of the N-quantiles defined is then set to 1/(N-1).

We compose our matched general population with survival data using the same calendar year than the study entry time of the RCT. For instance, if the study recruitment has been initiated in April 1999, the survival data for each wanted subgroup of the general population is extracted for the year 1999. By doing this, we perform a period analysis instead of a cohort analysis; in order to provide more sensible predicted estimates (27).

The average number of years lived during year t, for people dying during that year, is assumed to be 0.5. Using this half-cycle correction, the numbers of person alive between times t and t+1 for each subgroup G of persons having the same characteristics, $r_{general,t+1\left|t,\right.G}$, are equal to the following:

$$r_{general,t+1\left|t,\right.G}=ExposedtoRisk_{general,G,t}-0.5*Deaths_{general,G,t}$$

.

Where $ExposedtoRisk_{general,G,t}$ is the number of patients exposed to the risk of dying at time t for the subgroup G and $Deaths_{general,G,t}$ is the number of deaths between time t and t+1 for the subgroup G.

We refer to 1-year conditional survival probabilities: an interval-specific measure that gives the chance of surviving at time$t+1$, conditional on having survived to time $t$:$CS(t+1\left|t\right.)=S(t+1\left|t)/S(t)\right.$, whereby $S(t)$denotes the probability of survival up to time t > 0.

The survival probabilities at time t+1 years conditional on being alive at time t years, in the matched general population, $CS_{general,\left.t+1\right|t}$,are calculated as follows:

$$CS_{general,\left.t+1\right|t}=\sum_{G} CS_{general,G,\left.t+1\right|t}*W_{G}$$

Where$CS_{general,G,\left.t+1\right|t}$ is the survival probabilities at time t+1 years conditional on being alive at time t years in the subgroup G and $W_{G}$the weight of the subgroup G.

Their corresponding variances are equal to the equation below:

$$V(CS_{general,\left.t+1\right|t})=\sum_{G} \frac{CS_{general,G,\left.t+1\right|t}*(1-CS_{general,G,\left.t+1\right|t})}{r_{general,G,\left.t+1\right|t}}*W^{2}$$

The number of persons alive during the entire period, $r_{general,t+1\left|t\right.}$ and the number of persons at risk, $n_{general,t+1\left|t\right.}$, between times t and t+1, in the matched general population, are obtained by the following equations:

$n_{general,t+1\left|t\right.}=\frac{CS_{general,t+1\left|t\right.}*(1-CS_{general,t+1\left|t\right.})}{V(CS_{general,t+1\left|t\right.})}$.

$$r_{general,t+1\left|t\right.}=CS_{general,t+1\left|t\right.}*n_{general,t+1\left|t\right.}$$

Carrying this out generates a numerator of 1660 survivors at *t=*40

$$r_{general,\left.40\right|39}=1,660$$

$$S_{general,t+1}=CS_{general,t+1\left|t\right.}*S_{general,t}$$

Carrying this out generates a survival probability of 1.045% at *t=*40

$$n_{general,t+1}=r_{general,t+1\left|t\right.}/S_{general,t+1}$$

Carrying this out generates a denominator of 158,858 at risk at *t=*0

$$n_{general,0}=158,858$$
