# Supplementary material for: Extrapolation of Survival Curves from Cancer Trials Using External Information
Source: Med Decis Making. 2016 Sep 29;37(4):353–66. doi: 10.1177/0272989X16670604 (PMC6190619; doi:10.1177/0272989X16670604)
Supplement: Supplementary material [file Extrapolation_Appendix_B_v10_online_supp.docx]

## Appendix B. Construction of a head and neck cancer cohort matched to the Bonner trial population

Relative conditional survival probabilities and numbers at risk were extracted from the SEER database for each of the primary cancer sites reported in the Bonner RCT. Then, the relative conditional survival probabilities for each site were multiplied by the age- and gender-matched general conditional survival to obtain conditional survival predictions, and finally pooled to form a site-weighted average using the cancer site information in the RCT. A period analysis of 5 years (1997-2002) was applied to extract data from the SEER database. Data were available from 1970 onwards; as a consequence, a follow-up of 26 years was available. We did not adjust for stage or treatment, as these factors were less often recorded in the database than primary sites. Figure 2A shows the 1-year conditional survival probabilities obtained for each primary site and for a primary site-matched SEER population. 1-year conditional survival increases until around 5-years, and then stabilizes before slowly falling. RCT results are consistent with this trend.

The conditional survival data is presented in Table B.1.

Table B.1. Matched SEER conditional survival data.

| Time t in years from randomization | Numerator | Denominator |
| --- | --- | --- |
| 6 | 325 | 358 |
| 7 | 285 | 308 |
| 8 | 198 | 221 |
| 9 | 143 | 167 |
| 10 | 122 | 133 |
| 11 | 105 | 117 |
| 12 | 91 | 100 |
| 13 | 86 | 94 |
| 14 | 87 | 98 |
| 15 | 75 | 85 |
| 16 | 93 | 101 |
| 17 | 47 | 52 |
| 18 | 47 | 54 |
| 19 | 34 | 41 |
| 20 | 40 | 44 |
| 21 | 22 | 27 |
| 22 | 17 | 20 |
| 23 | 24 | 31 |
| 24 | 22 | 26 |
| 25 | 25 | 29 |
| 26 | 23 | 28 |

For the analyses including both SEER and general population conditional survival information, the general population numerator at 34 years was 23,080, and the denominator 30,196: representing an estimated 76.4% 1-year conditional survival.
