# Supplementary material for: Extrapolation of Survival Curves from Cancer Trials Using External Information
Source: Med Decis Making. 2016 Sep 29;37(4):353–66. doi: 10.1177/0272989X16670604 (PMC6190619; doi:10.1177/0272989X16670604)
Supplement: Supplementary material [file Extrapolation_Appendix_C_v10_online_supp.docx]

## Appendix C. Cubic Splines

We fit a cubic spline model to the log-cumulative hazard, where all parameters except the intercept,$\alpha$, depend on treatment $k$ (1=treatment, 0 = control)*.* There is therefore a spline for the control arm, and an additional spline for the relative treatment effect on the log-cumulative-hazard scale. All patients are assumed to start the RCT with the same risk of death.

$$x=\ln(t), t \text{in} \text{months}$$

$\ln(H_{k}(t))=s(x,\alpha,\gamma_{k})=\alpha+\gamma_{k,1}x+\gamma_{k,2}\nu_{1}(x)+...+\gamma_{k,m+1}\nu_{m}(x)$ with $k=0$for the control arm and $k=1$for the treatment arm

The $j-$ th basis function is defined for $j=1,...,m$ as

$$v_{j}(x)=(x-L_{j})_{+}^{3}-\lambda_{j}(x-L_{\min})_{+}^{3}-(1-\lambda_{j})(x-L_{\max})_{+}^{3}$$

and $\lambda_{j}=\frac{L_{\max}-L_{j}}{L_{\max}-L_{\min}}$

$(x-a)_{+}=\max(0,x-a)$.

Boundary knots, $L_{\min}$ and $L_{\max}$, and internal knots, $L_{1}$and $L_{2}$were placed at the same location for each arm. $L_{\min}$ was set equal to the log-time of the first death in the RCT data, i.e. log(1)=0. $L_{\max}$ was set equal to the log-time of the last assumed death in the general population data, 60 years, log(720)=6.58 (time in months). The first internal knot $k_{1}$ was placed in the middle of the RCT period i.e. log(30)=3.39 and the second internal knot $k_{2}$ was chosen approximately half way through the external data period on the log scale i.e. log(187)=5.23.
