# Supplementary material for: Extrapolation of Survival Curves from Cancer Trials Using External Information
Source: Med Decis Making. 2016 Sep 29;37(4):353–66. doi: 10.1177/0272989X16670604 (PMC6190619; doi:10.1177/0272989X16670604)
Supplement: Supplementary material [file Extrapolation_Appendix_D_v10_online_supp.docx]

## Appendix D. Winbugs code and data

The cubic splines model was defined as in Royston-Lambert 2011 [55].

***# Winbugs model***

model {

*##Definition of the knots*

lambda1<-(kmax-k1)/(kmax-kmin)

lambda2<-(kmax-k2)/(kmax-kmin)

*##Cubic splines model*

C<-10

for (i in 1:N) {

x[i]<-log(t[i])

*##Definition of the splines variables*

rcs1[i]<-x[i]

rcs2[i]<-pow(max(0,x[i]-k1),3)-lambda1*pow(max(0,x[i]-kmin),3)-(1-lambda1)*pow(max(0,x[i]-kmax),3)

rcs3[i]<-pow(max(0,x[i]-k2),3)-lambda2*pow(max(0,x[i]-kmin),3)-(1-lambda2)*pow(max(0,x[i]-kmax),3)

*##Definition of the derivatives of the splines variables*

drcs1[i]<-1

drcs2[i]<-3*pow(max(0,x[i]-k1),2)-3*lambda1*pow(max(0,x[i]-kmin),2)-3*(1-lambda1)*pow(max(0, x[i]-kmax),2)

drcs3[i]<-3*pow(max(0,x[i]-k2),2)-3*lambda2*pow(max(0,x[i]-kmin),2)-3*(1-lambda2)*pow(max(0, x[i]-kmax),2)

*## Restricted cubic splines functions are not listed under the standard distributions in Winbugs. However, the “zeros trick” describes in the Winbugs manual allows arbitrary sampling distributions to be used*

zeros[i]<-0

*## eta[i] is the linear predictor which includes the intercept gamma[1] and 3 further parameters* *gamma[2], gamma[3], gamma[4] for the control arm of the 3 spline variables rcs1, rcs2 and rcs3 and 3 further parameters* *beta[1], beta[2], beta[3] for* *the treatment arm of the 3 spline variables of rcs1, rcs2 and rcs 3.*

eta[i]<-gamma[1]+gamma[2]*rcs1[i]*(1-treat[i])+gamma[3]*rcs2[i]*(1-treat[i])+gamma[4]*rcs3[i]*(1-treat[i])+beta[1]*treat[i]*rcs1[i]+beta[2]*treat[i]*rcs2[i]+beta[3]*treat[i]*rcs3[i]

*## d.spi[i] is the linear predictor for the derivative of the spline variables. This has the same parameters as in the linear predictor but uses the derivative variables drcs1, drcs2 and drcs3.*

d.sp[i]<-gamma[2]*drcs1[i]*(1-treat[i])+gamma[3]*drcs2[i]*(1-treat[i])+gamma[4]*drcs3[i]*(1-treat[i])+beta[1]*treat[i]*drcs1[i]+beta[2]*treat[i]*drcs.treat2[i]+beta[3]*treat[i]*drcs.treat3[i]

*## lnL[i] is the log-likelihood*

lnL[i]<- -(d[i]*log(max((1/t[i])*d.sp[i]*exp(eta[i]), 0.00001))-exp(eta[i])) + C

zeros[i]~dpois(lnL[i])

##Predicted survival of trial population at times of external data

for (i in 1:24) {

*##Definition of the splines variables at times of external data*

x.RCT.Text[i]<-log(T.ext[i])

rcs1.RCT.Text[i]<-x.RCT.Text[i]

rcs2.RCT.Text[i]<-pow(max(0,x.RCT.Text[i]-k1),3)-lambda1*pow(max(0,x.RCT.Text[i]-kmin),3)-(1-lambda1)*pow(max(0,x.RCT.Text[i]-kmax),3)

rcs3.RCT.Text[i]<-pow(max(0,x.RCT.Text[i]-k2),3)-lambda2*pow(max(0,x.RCT.Text[i]-kmin),3)-(1-lambda2)*pow(max(0,x.RCT.Text[i]-kmax),3)

*##Definition of the derivatives of the splines variables at times of external data*

drcs1.RCT.Text[i]<-1

drcs2.RCT.Text[i]<-3*pow(max(0,x.RCT.Text[i]-k1),2)-3*lambda1*pow(max(0,x.RCT.Text[i]-kmin),2)-3*(1-lambda1)*pow(max(0, x.RCT.Text[i]-kmax),2)

drcs3.RCT.Text[i]<-3*pow(max(0,x.RCT.Text[i]-k2),2)-3*lambda2*pow(max(0,x.RCT.Text[i]-kmin),2)-3*(1-lambda2)*pow(max(0, x.RCT.Text[i]-kmax),2)

*##Definition of the linear predictors at times of external data for each arm, c=control, t=treatment*

eta.RCT.Text.c[i]<-gamma[1] + gamma[2]*rcs1.RCT.Text[i] + gamma[3]*rcs2.RCT.Text[i]+ gamma[4]*rcs3.RCT.Text[i]

eta.RCT.Text.t[i]<-gamma[1] + beta[1] *rcs1.RCT.Text[i]+beta[2]*rcs2. RCT.Text[i]+

beta[3]*rcs3.RCT.Text[i]

*##Definition of the linear predictors at times of external data for each arm, c=control, t=treatment*

d.sp.RCT.Text.c[i]<-gamma[2]*drcs1.RCT.Text[i]+gamma[3]*drcs2.RCT.Text[i]+gamma[4]*drcs3.RCT.Text[i]

d.sp.RCT.Text.t[i]<-beta[1]*drcs1.RCT.Text[i]+beta[2]*drcs 2.RCT.Text[i]+beta[3]*drcs3.RCT.Text[i]

*##Definition of the hazard functions at times of external data for each arm, c=control, t=treatment*

h.RCT.Text.c[i]<-1/T.ext[i]*d.sp.RCT.Text.c[i]*exp(eta.RCT.Text.c[i])

h.RCT.Text.t[i]<-1/T.ext[i]*d.sp.RCT.Text.t[i]*exp(eta.RCT.Text.t[i])

*##Definition of the survival functions at times of external data for each arm, c=control, t=treatment*

S.RCT.Text.c[i]<-max(0.00001,min(exp(-exp(eta.RCT.Text.c[i])),0.99999))

S.RCT.Text.t[i]<-max(0.00001,min(exp(-exp(eta.RCT.Text.t[i])),0.99999))

}

for (i in 1:21)

{

*##Definition of the conditional survival function in the control arm at times of external data*

CS.RCT.c[i]<-max(0.00001,min(S.RCT.Text.c[i+1]/S.RCT.Text.c[i],0.99999))

*##Model for the SEER conditional survival data*

r.b[i] ~ dbin (CS.ext.b[i],n.b[i])

*## Constraint using the SEER conditional survival data*

CS.ext.b[i]<-max(0.00001,min(CS.RCT.c[i],0.99999))

}

for (i in 1:1){

*##Model for the general population conditional survival data*

r.GP[i] ~ dbin (CS.GP[i],n.GP[i])

*##Constraint using the general population conditional survival data*

CS.RCT.c[i+21]<-max(0.00001,min(S.RCT.Text.c[(24)]/S.RCT.Text.c[(23)],0.99999))CS.GP[i]<-max(0.00001,min(CS.RCT.c[i+21],0.99999))

}

for (i in 1:23)

{

*##Model for the external hazard ratio data*

HR.ext.m[i]~dnorm(HR[i],HR.prec[i])

*##Constraint using the external hazard ratio data*

HR[i]<-h.RCT.Text.t[i+1]/h.RCT.Text.c[i+1]

}

*#Priors for the 4 gamma parameters*

for (j in 1:4){

gamma[j]~dnorm(0,0.01)

}

*#Priors for the 3 beta parameters*

for (j in 1:3) {

beta[j]~dnorm(0,0.01)

}

*# To compute results*

for (i in 1:68) {

x.pred[i]<-log(t.annual[i])

rcs1.pred[i]<-x.pred[i]

rcs2.pred[i]<-pow(max(0,x.pred[i]-k1),3)-lambda1*pow(max(0,x.pred[i]-kmin),3)-(1-lambda1)*pow(max(0,x.pred[i]-kmax),3)

rcs3.pred[i]<-pow(max(0,x.pred[i]-k2),3)-lambda2*pow(max(0,x.pred[i]-kmin),3)-(1-lambda2)*pow(max(0,x.pred[i]-kmax),3)

drcs1.pred[i]<-1

drcs2.pred[i]<-3*pow(max(0,x.pred[i]-k1),2)-3*lambda1*pow(max(0,x.pred[i]-kmin),2)-3*(1-lambda1)*pow(max(0, x.pred[i]-kmax),2)

drcs3.pred[i]<-3*pow(max(0,x.pred[i]-k2),2)-3*lambda2*pow(max(0,x.pred[i]-kmin),2)-3*(1-lambda2)*pow(max(0, x.pred[i]-kmax),2)

eta.pred.c[i]<-gamma[1]+gamma[2]*rcs1.pred[i]+gamma[3]*rcs2.pred[i]+gamma[4]*rcs3.pred[i]

eta.pred.t[i]<-gamma[1]+ beta[1] *rcs1.pred[i]+ beta[2]*rcs.treat2.pred[i]+ beta[3]*rcs.treat3.pred[i]

d.sp.pred.c[i]<-gamma[2]*drcs1.pred[i]+gamma[3]*drcs2.pred[i]+gamma[4]*drcs3.pred[i]

d.sp.pred.t[i]<-beta[1]*drcs1.pred[i]+beta[2]*drcs.treat2.pred[i]+beta[3]*drcs.treat3.pred[i]

h.c[i]<-1/t.annual[i]*d.sp.pred.c[i]*exp(eta.pred.c[i])

h.t[i]<-1/t.annual[i]*d.sp.pred.t[i]*exp(eta.pred.t[i])

*# Predicted annual hazard ratio*

HR.a[i]<-h.t[i]/h.c[i]

*# Predicted annual survival*

S.c[i]<-exp(-exp(eta.pred.c[i]))

S.t[i]<-exp(-exp(eta.pred.t[i]))

}

*# Predicted annual conditional survival*

for (i in 2:68) {

CS.c[i]<-S.c[i]/max(S.c[i-1],0.000001)

CS.t[i]<-S.t[i]/max(S.t[i-1],0.00000001)

}

*# Predicted mean in each arm, c=control, t=treatment*

mean.surv.c<-Mean3k(endL,nsubdiv,gamma[1],gamma[2],gamma[3],gamma[4],kmin,kmax,k1,k2)

mean.surv.t<-Mean3k(endL,nsubdiv,gamma[1],beta[1],beta[2],beta[3],kmin,kmax.treat,k.treat1,k.treat2)

*# Predicted mean difference*

diff.mean<-mean.surv.t-mean.surv.c

}

***# Data used***

list(N=424,nsubdiv=1000, endL=900, kmin=0, kmax=6.58, k1=3.39, k2=5.23, T.ext=c(60,72,84,96,108,120,132,144,156,168,180,192,204,216,228,240,252,264,276,288,300,312,

408, #Note this time-point is 1-year prior to 1st GP CS data-point

420),

r.GP=c(23080),

n.GP=c(30196),

n.b=c(358,308,221,167,133,117,100,94,98,85,101,52,54,41,44,27,20,31,26,29,28), r.b=c(325,285,198,143,122,105,91,86,87,75,93,47,47,34,40,22,17,24,22,25,23), HR.ext.m=c(1,1,1,1,1, 1,1,1,1,1, 1,1,1,1,1, 1,1,1,1,1, 1,1,1), HR.prec=c(100,100,100,100,100, 100,100,100,100,100, 100,100,100,100,100, 100,100,100,100,100, 100,100,100))

# IPD reconstructed from the KM curves

| t[] | d[] | treat[] |
| --- | --- | --- |
| 1 | 1 | 0 |
| 1 | 1 | 0 |
| 1.93 | 1 | 0 |
| 1.93 | 1 | 0 |
| 2.54 | 1 | 0 |
| 2.54 | 1 | 0 |
| 2.54 | 1 | 0 |
| 3.77 | 1 | 0 |
| 3.77 | 1 | 0 |
| 3.77 | 1 | 0 |
| 4.31 | 1 | 0 |
| 4.31 | 1 | 0 |
| 4.47 | 1 | 0 |
| 4.93 | 1 | 0 |
| 4.93 | 1 | 0 |
| 5.31 | 1 | 0 |
| 5.31 | 1 | 0 |
| 5.31 | 1 | 0 |
| 5.54 | 1 | 0 |
| 5.54 | 1 | 0 |
| 5.54 | 1 | 0 |
| 5.54 | 1 | 0 |
| 5.54 | 1 | 0 |
| 6.24 | 1 | 0 |
| 6.24 | 1 | 0 |
| 6.39 | 1 | 0 |
| 6.39 | 1 | 0 |
| 6.39 | 1 | 0 |
| 6.39 | 1 | 0 |
| 6.62 | 1 | 0 |
| 6.62 | 1 | 0 |
| 6.62 | 1 | 0 |
| 7.01 | 1 | 0 |
| 7.01 | 1 | 0 |
| 7.32 | 1 | 0 |
| 7.32 | 1 | 0 |
| 7.32 | 1 | 0 |
| 7.85 | 1 | 0 |
| 7.85 | 1 | 0 |
| 7.85 | 1 | 0 |
| 8.39 | 1 | 0 |
| 8.39 | 1 | 0 |
| 8.39 | 1 | 0 |
| 8.39 | 1 | 0 |
| 10 | 1 | 0 |
| 10 | 1 | 0 |
| 10.3 | 1 | 0 |
| 10.3 | 1 | 0 |
| 10.3 | 1 | 0 |
| 10.3 | 1 | 0 |
| 10.7 | 1 | 0 |
| 11.4 | 1 | 0 |
| 11.4 | 1 | 0 |
| 12.3 | 1 | 0 |
| 12.3 | 1 | 0 |
| 12.3 | 1 | 0 |
| 12.8 | 1 | 0 |
| 13.8 | 1 | 0 |
| 13.8 | 1 | 0 |
| 14.3 | 1 | 0 |
| 14.3 | 1 | 0 |
| 14.3 | 1 | 0 |
| 15 | 1 | 0 |
| 15 | 1 | 0 |
| 15 | 1 | 0 |
| 15.9 | 1 | 0 |
| 15.9 | 1 | 0 |
| 16.6 | 1 | 0 |
| 16.6 | 1 | 0 |
| 17.1 | 1 | 0 |
| 17.1 | 1 | 0 |
| 17.1 | 1 | 0 |
| 17.6 | 1 | 0 |
| 17.6 | 1 | 0 |
| 18 | 1 | 0 |
| 18 | 1 | 0 |
| 18.4 | 1 | 0 |
| 18.4 | 1 | 0 |
| 18.4 | 1 | 0 |
| 19 | 1 | 0 |
| 19.6 | 1 | 0 |
| 20 | 1 | 0 |
| 20 | 1 | 0 |
| 20.6 | 1 | 0 |
| 20.6 | 1 | 0 |
| 20.7 | 1 | 0 |
| 20.7 | 1 | 0 |
| 20.7 | 1 | 0 |
| 20.7 | 1 | 0 |
| 22.6 | 1 | 0 |
| 23.4 | 1 | 0 |
| 24.1 | 1 | 0 |
| 24.9 | 1 | 0 |
| 24.9 | 1 | 0 |
| 24.9 | 1 | 0 |
| 24.9 | 1 | 0 |
| 25.3 | 1 | 0 |
| 25.3 | 1 | 0 |
| 27.4 | 1 | 0 |
| 27.4 | 1 | 0 |
| 27.4 | 1 | 0 |
| 29.1 | 1 | 0 |
| 29.6 | 1 | 0 |
| 29.6 | 1 | 0 |
| 30.9 | 1 | 0 |
| 30.9 | 1 | 0 |
| 30.9 | 1 | 0 |
| 31.7 | 1 | 0 |
| 31.7 | 1 | 0 |
| 32.2 | 1 | 0 |
| 34.4 | 1 | 0 |
| 36.3 | 1 | 0 |
| 40 | 1 | 0 |
| 40 | 1 | 0 |
| 42.6 | 1 | 0 |
| 43 | 1 | 0 |
| 47.4 | 1 | 0 |
| 50 | 1 | 0 |
| 51.4 | 1 | 0 |
| 51.4 | 1 | 0 |
| 52.5 | 1 | 0 |
| 53.5 | 1 | 0 |
| 58.4 | 1 | 0 |
| 59.5 | 1 | 0 |
| 59.4 | 1 | 0 |
| 1.465 | 0 | 0 |
| 2.235 | 0 | 0 |
| 3.425 | 0 | 0 |
| 5.12 | 0 | 0 |
| 6.315 | 0 | 0 |
| 7.585 | 0 | 0 |
| 8.89 | 0 | 0 |
| 12.55 | 0 | 0 |
| 14.65 | 0 | 0 |
| 17.35 | 0 | 0 |
| 23 | 0 | 0 |
| 26.85 | 0 | 0 |
| 30.45 | 0 | 0 |
| 31.3 | 0 | 0 |
| 31.95 | 0 | 0 |
| 33.3 | 0 | 0 |
| 33.3 | 0 | 0 |
| 33.3 | 0 | 0 |
| 33.3 | 0 | 0 |
| 35.35 | 0 | 0 |
| 35.35 | 0 | 0 |
| 35.35 | 0 | 0 |
| 37.3 | 0 | 0 |
| 37.3 | 0 | 0 |
| 37.3 | 0 | 0 |
| 37.3 | 0 | 0 |
| 39.15 | 0 | 0 |
| 39.15 | 0 | 0 |
| 40.55 | 0 | 0 |
| 40.55 | 0 | 0 |
| 41.85 | 0 | 0 |
| 41.85 | 0 | 0 |
| 41.85 | 0 | 0 |
| 42.8 | 0 | 0 |
| 45.05 | 0 | 0 |
| 45.05 | 0 | 0 |
| 45.05 | 0 | 0 |
| 45.05 | 0 | 0 |
| 45.05 | 0 | 0 |
| 45.05 | 0 | 0 |
| 45.05 | 0 | 0 |
| 45.05 | 0 | 0 |
| 45.05 | 0 | 0 |
| 47.25 | 0 | 0 |
| 48.7 | 0 | 0 |
| 48.7 | 0 | 0 |
| 48.7 | 0 | 0 |
| 48.7 | 0 | 0 |
| 48.7 | 0 | 0 |
| 50.9 | 0 | 0 |
| 50.9 | 0 | 0 |
| 51.75 | 0 | 0 |
| 52.3 | 0 | 0 |
| 52.8 | 0 | 0 |
| 53.3 | 0 | 0 |
| 55.8 | 0 | 0 |
| 55.8 | 0 | 0 |
| 55.8 | 0 | 0 |
| 55.8 | 0 | 0 |
| 55.8 | 0 | 0 |
| 55.8 | 0 | 0 |
| 55.8 | 0 | 0 |
| 55.8 | 0 | 0 |
| 58.25 | 0 | 0 |
| 58.95 | 0 | 0 |
| 59.45 | 0 | 0 |
| 62.55 | 0 | 0 |
| 62.55 | 0 | 0 |
| 62.55 | 0 | 0 |
| 62.55 | 0 | 0 |
| 62.55 | 0 | 0 |
| 62.55 | 0 | 0 |
| 65.1 | 0 | 0 |
| 65.1 | 0 | 0 |
| 65.1 | 0 | 0 |
| 65.1 | 0 | 0 |
| 65.1 | 0 | 0 |
| 65.1 | 0 | 0 |
| 65.1 | 0 | 0 |
| 65.1 | 0 | 0 |
| 65.1 | 0 | 0 |
| 65.1 | 0 | 0 |
| 65.1 | 0 | 0 |
| 65.1 | 0 | 0 |
| 65.1 | 0 | 0 |
| 65.1 | 0 | 0 |
| 65.1 | 0 | 0 |
| 65.1 | 0 | 0 |
| 0.417 | 1 | 1 |
| 1.11 | 1 | 1 |
| 1.46 | 1 | 1 |
| 1.63 | 1 | 1 |
| 2.32 | 1 | 1 |
| 2.32 | 1 | 1 |
| 2.67 | 1 | 1 |
| 3.02 | 1 | 1 |
| 3.36 | 1 | 1 |
| 3.54 | 1 | 1 |
| 4.06 | 1 | 1 |
| 5.1 | 1 | 1 |
| 5.27 | 1 | 1 |
| 5.62 | 1 | 1 |
| 5.79 | 1 | 1 |
| 5.96 | 1 | 1 |
| 6.31 | 1 | 1 |
| 6.31 | 1 | 1 |
| 6.48 | 1 | 1 |
| 7 | 1 | 1 |
| 7.18 | 1 | 1 |
| 7.18 | 1 | 1 |
| 7.18 | 1 | 1 |
| 7.7 | 1 | 1 |
| 8.22 | 1 | 1 |
| 8.39 | 1 | 1 |
| 8.39 | 1 | 1 |
| 8.74 | 1 | 1 |
| 8.74 | 1 | 1 |
| 8.74 | 1 | 1 |
| 8.91 | 1 | 1 |
| 9.08 | 1 | 1 |
| 9.6 | 1 | 1 |
| 10.1 | 1 | 1 |
| 10.1 | 1 | 1 |
| 10.3 | 1 | 1 |
| 10.6 | 1 | 1 |
| 10.6 | 1 | 1 |
| 10.8 | 1 | 1 |
| 11.2 | 1 | 1 |
| 11.3 | 1 | 1 |
| 11.3 | 1 | 1 |
| 11.5 | 1 | 1 |
| 11.5 | 1 | 1 |
| 11.9 | 1 | 1 |
| 11.9 | 1 | 1 |
| 11.9 | 1 | 1 |
| 12 | 1 | 1 |
| 12 | 1 | 1 |
| 12.4 | 1 | 1 |
| 12.4 | 1 | 1 |
| 12.4 | 1 | 1 |
| 12.4 | 1 | 1 |
| 12.4 | 1 | 1 |
| 12.5 | 1 | 1 |
| 12.7 | 1 | 1 |
| 13.2 | 1 | 1 |
| 13.2 | 1 | 1 |
| 14.3 | 1 | 1 |
| 14.5 | 1 | 1 |
| 15 | 1 | 1 |
| 16 | 1 | 1 |
| 16.2 | 1 | 1 |
| 17.2 | 1 | 1 |
| 17.6 | 1 | 1 |
| 18.1 | 1 | 1 |
| 18.4 | 1 | 1 |
| 18.8 | 1 | 1 |
| 19.3 | 1 | 1 |
| 19.7 | 1 | 1 |
| 20 | 1 | 1 |
| 20.3 | 1 | 1 |
| 20.3 | 1 | 1 |
| 20.5 | 1 | 1 |
| 20.9 | 1 | 1 |
| 22.1 | 1 | 1 |
| 22.4 | 1 | 1 |
| 22.8 | 1 | 1 |
| 22.9 | 1 | 1 |
| 24.7 | 1 | 1 |
| 25.4 | 1 | 1 |
| 25.9 | 1 | 1 |
| 26.9 | 1 | 1 |
| 27.8 | 1 | 1 |
| 28.5 | 1 | 1 |
| 29.5 | 1 | 1 |
| 29.5 | 1 | 1 |
| 30.2 | 1 | 1 |
| 31.1 | 1 | 1 |
| 33.2 | 1 | 1 |
| 34.2 | 1 | 1 |
| 35.6 | 1 | 1 |
| 35.8 | 1 | 1 |
| 36.1 | 1 | 1 |
| 36.3 | 1 | 1 |
| 44.8 | 1 | 1 |
| 45.3 | 1 | 1 |
| 47.2 | 1 | 1 |
| 47.6 | 1 | 1 |
| 47.7 | 1 | 1 |
| 48.9 | 1 | 1 |
| 49.5 | 1 | 1 |
| 53.3 | 1 | 1 |
| 55.5 | 1 | 1 |
| 56.7 | 1 | 1 |
| 58.8 | 1 | 1 |
| 59.7 | 1 | 1 |
| 5.01 | 0 | 1 |
| 11.95 | 0 | 1 |
| 14 | 0 | 1 |
| 15.9 | 0 | 1 |
| 18 | 0 | 1 |
| 22.5 | 0 | 1 |
| 24.95 | 0 | 1 |
| 27.4 | 0 | 1 |
| 31 | 0 | 1 |
| 31.9 | 0 | 1 |
| 32.75 | 0 | 1 |
| 33.6 | 0 | 1 |
| 34.5 | 0 | 1 |
| 35.5 | 0 | 1 |
| 36.4 | 0 | 1 |
| 38.35 | 0 | 1 |
| 38.35 | 0 | 1 |
| 38.35 | 0 | 1 |
| 40.65 | 0 | 1 |
| 40.65 | 0 | 1 |
| 40.65 | 0 | 1 |
| 42.8 | 0 | 1 |
| 42.8 | 0 | 1 |
| 42.8 | 0 | 1 |
| 42.8 | 0 | 1 |
| 42.8 | 0 | 1 |
| 42.8 | 0 | 1 |
| 42.8 | 0 | 1 |
| 42.8 | 0 | 1 |
| 42.8 | 0 | 1 |
| 42.8 | 0 | 1 |
| 42.8 | 0 | 1 |
| 44.9 | 0 | 1 |
| 45.2 | 0 | 1 |
| 45.4 | 0 | 1 |
| 45.9 | 0 | 1 |
| 46.1 | 0 | 1 |
| 46.4 | 0 | 1 |
| 46.8 | 0 | 1 |
| 47.1 | 0 | 1 |
| 47.5 | 0 | 1 |
| 47.8 | 0 | 1 |
| 48 | 0 | 1 |
| 48.3 | 0 | 1 |
| 48.7 | 0 | 1 |
| 49 | 0 | 1 |
| 49.4 | 0 | 1 |
| 49.7 | 0 | 1 |
| 50.4 | 0 | 1 |
| 50.6 | 0 | 1 |
| 51.8 | 0 | 1 |
| 51.8 | 0 | 1 |
| 51.8 | 0 | 1 |
| 51.8 | 0 | 1 |
| 51.8 | 0 | 1 |
| 51.8 | 0 | 1 |
| 51.8 | 0 | 1 |
| 53 | 0 | 1 |
| 53.35 | 0 | 1 |
| 53.7 | 0 | 1 |
| 53.9 | 0 | 1 |
| 54.2 | 0 | 1 |
| 54.6 | 0 | 1 |
| 54.9 | 0 | 1 |
| 55.1 | 0 | 1 |
| 55.45 | 0 | 1 |
| 55.8 | 0 | 1 |
| 56.1 | 0 | 1 |
| 56.3 | 0 | 1 |
| 56.65 | 0 | 1 |
| 57 | 0 | 1 |
| 57.2 | 0 | 1 |
| 57.5 | 0 | 1 |
| 57.9 | 0 | 1 |
| 58.2 | 0 | 1 |
| 58.4 | 0 | 1 |
| 58.7 | 0 | 1 |
| 59.1 | 0 | 1 |
| 59.4 | 0 | 1 |
| 59.6 | 0 | 1 |
| 60.65 | 0 | 1 |
| 63.65 | 0 | 1 |
| 63.65 | 0 | 1 |
| 63.65 | 0 | 1 |
| 63.65 | 0 | 1 |
| 63.65 | 0 | 1 |
| 63.65 | 0 | 1 |
| 63.65 | 0 | 1 |
| 66.3 | 0 | 1 |
| 66.3 | 0 | 1 |
| 66.3 | 0 | 1 |
| 66.3 | 0 | 1 |
| 66.3 | 0 | 1 |
| 66.3 | 0 | 1 |
| 66.3 | 0 | 1 |
| 66.3 | 0 | 1 |
| 66.3 | 0 | 1 |
| 66.3 | 0 | 1 |
| 66.3 | 0 | 1 |
| 66.3 | 0 | 1 |
| 66.3 | 0 | 1 |
| 66.3 | 0 | 1 |
| 66.3 | 0 | 1 |
| 66.3 | 0 | 1 |
| END |  |  |

***Subroutine Mean3k called by the WinBUGS program above***

*Blue color* ***represents changes from the blackbox module template***

MODULE WBDev*Mean3k*;

IMPORT

WBDevVector,

WBDevScalar,

Math,

WBDevSpecfunc;

TYPE

Function = POINTER TO RECORD (WBDevScalar.Node) END;

Factory = POINTER TO RECORD (WBDevScalar.Factory) END;

VAR

**fact**-: WBDevScalar.Factory;

PROCEDURE (func: Function) DeclareArgTypes (OUT args: ARRAY OF CHAR);

BEGIN

*#number of parameters=10*

args := "ssssssssss";

END DeclareArgTypes;

PROCEDURE (func: Function) Evaluate (OUT value: REAL);

CONST

*#Definition of the parameters*

timeupper = 0; nsubdiv = 1; gamma1 = 2; gamma2= 3; gamma3= 4; gamma4=5; kmin= 6; kmax= 7; k1= 8;k2=9;

VAR

*#Definition of the variables*

i,j,n: INTEGER;

t,h,a,b,c,kamin,kamax,ka1,x,lambda1, rcs1, rcs2,eta,xka1,xkamin,xkamax,d,ka2,xka2,lambda2,rcs3: REAL;

BEGIN

*#Definition of the function: the area under the curve is computed using the Simpson’s rule [60]. This rule is a method for numerical integration that provides numerical approximation of definite integrals.*

a := func.arguments[gamma1][0].Value();

b:= func.arguments[gamma2][0].Value();

c:= func.arguments[gamma3][0].Value();

d:= func.arguments[gamma4][0].Value();

kamin:= func.arguments[kmin][0].Value();

kamax:= func.arguments[kmax][0].Value();

ka1:= func.arguments[k1][0].Value();

ka2:= func.arguments[k2][0].Value();

n:= SHORT(ENTIER(func.arguments[nsubdiv][0].Value()));

t := func.arguments[timeupper][0].Value();

i:=0;

h:=t/n;

value:=1;

INC(i);

j:=0;

lambda1:=(kamax-ka1)/(kamax-kamin);

lambda2:=(kamax-ka2)/(kamax-kamin);

WHILE i < n DO;

x:=Math.Ln(i*h);

rcs1:=x;

IF (x-ka1>0) THEN;

xka1:=x-ka1;

ELSE;

xka1:=0;

END;

IF (x-ka2>0) THEN;

xka2:=x-ka2;

ELSE;

xka2:=0;

END;

IF (x-kamin>0) THEN;

xkamin:=x-kamin;

ELSE;

xkamin:=0;

END;

IF (x-kamax>0) THEN;

xkamax:=x-kamax;

ELSE;

xkamax:=0;

END;

rcs2:=WBDevSpecfunc.Power(xka1,3)-lambda1*WBDevSpecfunc.Power(xkamin,3)-(1-lambda1)*WBDevSpecfunc.Power(xkamax,3);

rcs3:=WBDevSpecfunc.Power(xka2,3)-lambda2*WBDevSpecfunc.Power(xkamin,3)-(1-lambda2)*WBDevSpecfunc.Power(xkamax,3);

eta:=a+b*rcs1+c*rcs2+d*rcs3;

IF (i=2*j+1) THEN;

value:= value + 4*(Math.Exp(eta-Math.Exp(eta)));

INC(j);

ELSE;

value:= value + 2*(Math.Exp(eta-Math.Exp(eta)));

END;

INC(i);

END;

x:=Math.Ln(t);

rcs1:=x;

IF ((x-ka1)>0) THEN;

xka1:=x-ka1;

ELSE;

xka1:=0;

END;

IF (x-ka2>0) THEN;

xka2:=x-ka2;

ELSE;

xka2:=0;

END;

IF ((x-kamin)>0) THEN;

xkamin:=x-kamin;

ELSE;

xkamin:=0;

END;

IF ((x-kamax)>0) THEN;

xkamax:=x-kamax;

ELSE;

xkamax:=0;

END;

rcs2:=WBDevSpecfunc.Power(xka1,3)-lambda1*WBDevSpecfunc.Power(xkamin,3)-(1-lambda1)*WBDevSpecfunc.Power(xkamax,3);

rcs3:=WBDevSpecfunc.Power(xka2,3)-lambda2*WBDevSpecfunc.Power(xkamin,3)-(1-lambda2)*WBDevSpecfunc.Power(xkamax,3);

eta:=a+b*rcs1+c*rcs2+d*rcs3;

value:= value + Math.Exp(eta-Math.Exp(eta));

value:= (t/n)*value/3

END Evaluate;

PROCEDURE (f: Factory) New (option: INTEGER): Function;

VAR

func: Function;

BEGIN

NEW(func); func.Initialize; **RETURN** func;

END New;

PROCEDURE **Install***;

BEGIN

WBDevScalar.Install(fact);

END Install;

PROCEDURE Init;

VAR

f: Factory;

BEGIN

NEW(f); fact := f;

END Init;

BEGIN

Init;

END WBDevMean3k.
