# Supplementary material for: Extrapolation of Survival Curves from Cancer Trials Using External Information
Source: Med Decis Making. 2016 Sep 29;37(4):353–66. doi: 10.1177/0272989X16670604 (PMC6190619; doi:10.1177/0272989X16670604)
Supplement: Supplementary material [file Extrapolation_Appendix_E_v10_online_supp.docx]

## Appendix E: Initial values, convergence, and posterior sampling

We assessed convergence by running multiple chains with different initial values, then inspecting history plots, density plots for key model outputs (survival estimates) and the gelman-rubin statistics.

For the model based on RCT data alone, we present results based on a burn-in period of 20,000 simulations, and posterior inference was based on 350,000 samples for the splines model, 150,000 for the AFT log-normal model, and 300,000 for the generalised gamma model.

For the constrained models, we present results based on a burn-in period of 20,000 simulations, and posterior inference was based on 70,000 samples for the splines model, 30,000 for the AFT log-normal model, and 60,000 for the generalised gamma model.

Convergence was problematic in some cases, and care was required in the choice of initial values to avoid the chain sampling from infeasible curves (see below).

*Initial values*

Spline function parameters of splines have no direct interpretation, making it difficult to choose a realistic set of initial values. Furthermore, due to the extremely flexible nature of the spline functions, convergence can be problematic if there is not sufficient data to determine the exact shape of the curves between the knots, and especially in the tail of the curves. This is essentially a problem of identifiability. We found that some choices of initial value found a solution that gave infeasible predicted survival curves, especially when we did not include all evidence sources. In such cases it is often suggested to use the Maximum Likelihood Estimate (MLE) to choose initial values [56], however due to the complexity of the relationships between our evidence and the model, it is not possible to evaluate the MLE. Instead we used the following strategy to identify realistic initial values:

1. Run the model with multiple chains of initial values.
2. Choose the chain with the minimum Dbar (i.e. the chains that fit the data best, and closest to MLE),
3. We then used descriptive statistics from the joint distribution from the chain with minimum Dbar to identify new sets of initial values (covering the full range of jointly plausible values)
4. Repeat steps 1-3 until Dbar seems to stabilise, i.e. when Dbar is not decreasing anymore after a reasonable number of attempts.

The sets of initial values identified are then used to check for convergence and to obtain results, as described above.
